# Different Impacts of MucR Binding to the *babR* and *virB* Promoters on Gene Expression in *Brucella abortus* 2308

**DOI:** 10.3390/biom10050788

**Published:** 2020-05-19

**Authors:** Giorgia Borriello, Veronica Russo, Rubina Paradiso, Marita Georgia Riccardi, Daniela Criscuolo, Gaetano Verde, Rosangela Marasco, Paolo Vincenzo Pedone, Giorgio Galiero, Ilaria Baglivo

**Affiliations:** 1 Experimental Zooprophylactic Institute of Southern Italy, via Salute, 2, 80055 Portici, Italy; giorgia.borriello@izsmportici.it (G.B.); rubina.paradiso@izsmportici.it (R.P.); maritageorgia.riccardi@izsmportici.it (M.G.R.); daniela.criscuolo@izsmportici.it (D.C.); 2 Department of Environmental, Biological and Pharmaceutical Sciences and Technologies, University of Campania “Luigi Vanvitelli”, via Vivaldi—43, 81100 Caserta, Italy; veronica.russo1@unicampania.it (V.R.); rosangela.marasco@unicampania.it (R.M.); paolov.pedone@unicampania.it (P.V.P.); 3 Institute of Genetics and Biophysics (IGB) “Adriano Buzzati-Traverso”, Consiglio Nazionale delle Ricerche (CNR), 80134 Naples, Italy; gaetanoverde13@gmail.com; 4 Flomics Biotech, Carrer Dr. Aiguader 88, 08003 Barcelona, Spain

**Keywords:** *Brucella abortus*, *virB* genes, MucR, BabR, H-NS, virulence gene expression regulation, virulence, *babR* promoter, *virB* promoter

## Abstract

The protein MucR from *Brucella abortus* has been described as a transcriptional regulator of many virulence genes. It is a member of the Ros/MucR family comprising proteins that control the expression of genes important for the successful interaction of α-proteobacteria with their eukaryotic hosts. Despite clear evidence of the role of MucR in repressing virulence genes, no study has been carried out so far demonstrating the direct interaction of this protein with the promoter of its target gene *babR* encoding a LuxR-like regulator repressing *virB* genes. In this study, we show for the first time the ability of MucR to bind the promoter of *babR* in electrophoretic mobility shift assays demonstrating a direct role of MucR in repressing this gene. Furthermore, we demonstrate that MucR can bind the *virB* gene promoter. Analyses by RT-qPCR showed no significant differences in the expression level of *virB* genes in *Brucella abortus* CC092 lacking MucR compared to the wild-type *Brucella abortus* strain, indicating that MucR binding to the *virB* promoter has little impact on *virB* gene expression in *B. abortus* 2308. The MucR modality to bind the two promoters analyzed supports our previous hypothesis that this is a histone-like protein never found before in *Brucella*.

## 1. Introduction

*Brucella* spp. are the bacteria responsible for one of the most widespread zoonoses worldwide. Infection from *Brucella abortus* and *Brucella melitensis* causes abortion in cattle, sheep and goats which constitutes a serious threat to the livestock industry and a source of human infection [1]. The vaccine strain RB51 can also be responsible for disease transmission to humans through pathogen excretion in milk [2,3]. For these reasons, studies of the virulence determinants of *Brucella* and the mechanisms that regulate the expression of virulence genes are fundamental to understand how to handle the spread of brucellosis. 

The protein MucR from *Brucella abortus* has been described as a transcriptional regulator of virulence genes [4]. Deletion of the gene encoding for MucR produces attenuated *Brucella* strains, confirming the pivotal role of this protein in virulence [4,5]. MucR is one of the members of the Ros/MucR protein family [4,6] which comprises prokaryotic zinc finger proteins like Ros from *Agrobacterium tumefaciens* [7,8] and Ml1, Ml2 and Ml3 (Mls) from *Mesorhizobium loti* [6,9]. All of the characterized members of the Ros/MucR protein family bind DNA [4,6,10,11] and the MucR homolog Ros from *Agrobacterium tumefaciens* represses some of the *vir* genes that are responsible for transferring this bacterium’s DNA into its plant host [10]. Recently, we have demonstrated that the Mls and MucR form higher-order oligomers and interact mostly with the DNA minor groove recognizing AT-rich DNA sequences containing T–A steps. These findings led us to suggest that members of the Ros/MucR family could play the same role in α-proteobacteria in regulating virulence gene expression as the histone-like proteins H-NS in β- and γ-proteobacteria [12,13,14]. In fact, H-NS proteins regulate virulence gene expression by binding AT-rich sequences containing T-A steps [15]. These AT-rich targets constitute nucleation sites from which H-NS proteins can extend their presence on the nucleoid by oligomerization [15,16]. One of the gene whose expression is controlled by MucR in *Brucella abortus* is *babR* which encodes the corresponding LuxR-type quorum sensing regulator BabR [17], also known as BlxR [18]. In bacteria interacting with eukaryotic hosts, quorum-sensing plays a central role for successful symbiotic or infectious interactions [19]. The precise physiologic role of BabR in *Brucella* is unknown, but it is thought to work in a reciprocal fashion with another LuxR-type quorum sensing regulator, VjbR, to properly coordinate the expression of *Brucella* virulence genes [20,21]. Notably, BabR represses the expression of the *virB* genes in *B. abortus* 2308 [22] while VjbR activates these genes [23]. The *virB* operon encodes the proteins that constitute the type IV secretion system (T4SS) which is necessary for a successful infection. The VirB proteins assemble in a complex through the bacterial envelope allowing the translocation of effector proteins out of the bacterial cell into the cytoplasm of the host cell [24,25]. In turn, these effector proteins disrupt the normal endolysosomal trafficking of the *Brucella*-containing vacuoles (BCVs) in host cells and allow the brucellae to reach a specialized intracellular compartment known as the replicative BCV where they maintain their intracellular persistence [26]. 

Despite clear evidence of the role played by MucR in repressing *babR* gene expression and in *Brucella* virulence [4,5], no study has been carried out so far about the ability of MucR to bind the promoters of *babR* and/or that of *virB* genes. Here we demonstrate that MucR from *Brucella abortus* binds the promoter of *babR* gene indicating its direct role in *babR* repression. Furthermore, we report that MucR can interact with the *virB* operon promoter as well, but we show that the binding affinity for this latter promoter is lower compared to that for *babR* promoter. In line with this difference in the affinity of MucR binding to the two promoters, transcriptional analysis suggests that MucR does not exert any impact on *virB* gene expression in *B. abortus* 2308 under the experimental conditions employed in this study. Our findings demonstrate for the first time a direct role of MucR in regulating the *babR* gene expression and suggest an indirect role of this protein in the expression of *virB* genes. The modality of MucR binding to the promoters analyzed still supports our previous hypothesis that MucR (and the other members of the Ros/MucR family) is the H-NS-like protein that has never been found before in many species of α-proteobacteria [27]. 

## 2. Materials and Methods 

### 2.1. Cloning, Protein Expression and Purification

The gene encoding the protein MucR was cloned in the pET-22b(+) vector (MucR-pet22b(+)) as previously reported [12]. The vector mucR-pet22b(+) was used to transform E. coli BL21DE3. Transformed colonies were inoculated in LB medium and grown until OD_600_ reached the value of 0.45. At this point of E. coli growth, the expression of the protein was induced adding IPTG at a final concentration of 1 M and carried out at 28 °C for 1 h. The purification of MucR was carried out as previously reported [12]. The protein eluted from a Mono S HR 5/5 cation exchange chromatography column in the 0.4–0.8 M NaCl concentration range.

### 2.2. Electrophoretic Mobility Shift Assay 

The electrophoretic mobility shift assay (EMSA) experiments were carried out as previously described [28]. In detail, protein amounts from 0.2 up to 1.6 μg were incubated for 10 min on ice with 5 pmol of the double-stranded oligonucleotides in binding buffer (25 mM HEPES pH 7.9, 50 mM KCl, 6.25 mM MgCl2, 5% glycerol). The reaction mixture of 20 μL was loaded onto a 5% polyacrylamide gel in 0.5X TBE and run at room temperature for 75 min at 200 V. Gels were then stained for 20 min with Diamond™ Nucleic Acid Dye (Promega), rinsed with milliQ water and imaged by Typhoon Trio+ scanner (GE Healthcare). In particular, the amount of MucR used in the EMSAs with babR60 was 0.2, 0.4 and 0.8 μg. In the EMSAs with all the other double-stranded oligonucleotides tested, the amount of MucR used was 0.4, 0.8 and 1.6 μg.

For competition assays, 5 pmol of the FAM-labeled double-stranded babR60 was used as a probe incubated for 10 min on ice with 0.4 μg of MucR. Then, 10X, 20X or 40X of each competitor was added and the reaction mixtures were incubated on ice for another 10 min. The electrophoreses were carried out as already describe above and the fluorescent signals were imaged by Typhoon Trio+ scanner (GE Healthcare).

### 2.3. RT-qPCR

RNA was isolated from *B. abortus* strains as previously described [22]. The CC092 strain is an isogenic mucR mutant originated as reported by Caswell et al. [4]. Briefly, *B. abortus* strains were grown to early stationary phase in brucella broth with constant shaking at 37 °C with 5% CO_2_. *B. abortus* cultures were then either allowed to continue to grow until late stationary for brucella broth (BB) treatments or pelleted and resuspended in equal volume of E-medium [29] and incubated for an additional 4 hours at 37 °C with 5% CO_2_. An equal volume of cold 1:1 acetone:ethanol was added to cultures for storage at −80 °C for up to one month. For RNA isolation, the cell/acetone:ethanol mixture was thawed and pelleted at 16,000× *g* for 3 min and RNA was isolated from cells using TRIzol (Invitrogen, Carlsbad, CA) and subsequent ethanol precipitation, as previously described [22].

Following RNA isolation, genomic DNA was removed from approximately 2 µg RNA using DNase I (Thermo Fisher Scientific, Waltham, MA) as previously described [22] and cDNA was generated from approximately 1 µg treated RNA using the SuperScript III First-Strand Synthesis System (Invitrogen, Carlsbad, CA). The following PCR conditions were used: 30 min at 37 °C, 20 min at 50 °C and 5 min at 85 °C. cDNA was then treated with RNase H for 20 min at 37 °C, diluted 1:10 in nuclease-free water and used for quantitative PCR. Briefly, 8 µL diluted cDNA was added to 10 µL 2X PowerUp SYBR Green Master Mix (Thermo Fisher Scientific, Waltham, MA) and 2 µL of 5 mM primer mix (i.e., forward and reverse). Gene-specific primers were generated for *B. abortus* 2308 *virB1* (5′ – TGGCAGCACCTACGCACAAG – 3′; 5′ – GCCTTGTTCCTGACCGGGCA – 3′), *virB2* (5′ – ACCATCGTTACCATAGCCATC – 3′; 5′ – GTAAGAGGCAATTTCGGCG – 3′) and *babR* (5′ – CACCAATCTCCTGCATCTATCC – 3′; 5′ – GCTGATGTGGAAATTGACCG – 3′). Each gene-specific reaction was normalized to reactions using primers specific for 16 s rRNA (5′ – TCTCACGACACGAGCTGACG-3′; 5′ – CGCAGAACCTTACCAGCCCT – 3′) and GAP (5′ – GCAGTTCGCGTCGCAAT – 3′; 5′ – GAAGATGTGCGTTGGTTTCG – 5′). All amplicon lengths were approximately 150 bp and all samples were run in triplicate on quantitative PCR plates. RT-qPCR conditions included 2 min at 50 °C and 2 min at 95 °C followed by 40 cycles of 3 sec at 95 °C and 30 sec at 60 °C. Fluorescence representing SYBR green incorporation was analyzed using a BioRad CFX96 Real-Time system (BioRad, Hercules, CA, USA) and relative abundances of mRNA were subsequently analyzed using the 2^(-delta delta Ct) method [30]. The results obtained by RT-qPCR experiments were confirmed by two biological and three technical replications.

### 2.4. Isolation of *B. abortus* Field Strains and Genomic Sequencing

Eighteen *Brucella abortus* isolates obtained from the lymph nodes of water buffaloes slaughtered within the Italian National Plan for the control of brucellosis were investigated. The animals included in this study were collected from 18 water buffalo herds located in southern Italy, in the Campania region. As indicated by the Italian Control Program, based on a test-and-slaughter approach, animals were not vaccinated and all the subjects that tested positive to serological tests (Rose Bengal and complement fixation tests) were culled and processed for microbiological isolation of *Brucella* spp. [31]. Bacterial isolates were characterized by biochemical and molecular tests for species and biovar identification by the National Reference Center for Animal Brucellosis [31]. For molecular analysis, strains were inoculated onto *Brucella* medium base (Oxoid) and incubated at 37 °C in 5%–10% CO_2._ DNA was extracted by QIAamp DNA mini kit (QIAGEN) following manufacturer’s protocol. For sequencing, we used approximately 50 to 100 ng of genomic DNA to produce fragments of 400 bp in length. Libraries were prepared for Ion Torrent sequencing platform. The generated sequencing reads were checked using FastQC and low-quality sequences were removed by using PRINSEQ lite. High-quality sequences were successfully assembled using SPAdes [32] version 3.8.2, improved with Pilon 1.8 and manually checked to close eventual gaps. The draft assemblies of the 18 strains were annotated using the NCBI Prokaryotic Genome Annotation Pipeline version 4.2.

The sequences of the *virB* genes of the 18 *Brucella* strains included in this study were compared by using the blast tool (https://blast.ncbi.nlm.nih.gov/Blast.cgi?PAGE_TYPE=BlastSearch&BLAST_SPEC=blast2seq&LINK_LOC=align2seq) to identify differences in nucleotide sequence in comparison with the reference strain *B. abortus* 2308 (chromosome I GenBank accession: AM040264.1; chromosome II GenBank accession: AM040265.1).

The presence of the identified mutations in *virB* genes was verified in 68 *B. abortus* field strains belonging to the Experimental Zooprophylactic Institute of Southern Italy bacterial collection by DNA sequencing. For this purpose, bacterial strains were cultured and processed for DNA extraction as described above. Amplifications were performed in a final volume of 25 μL including 1.5 U of Platinum® Taq DNA Polymerase (Invitrogen, Life Technologies), 1X PCR Buffer with 1.5 mM MgCl_2_, 0.2 mM each DNTPs, 0.4 μM of primer forward, 0.4 μM of primer reverse and 3 μL of DNA. The primers for *virB* gene amplification were designed using the online tool Primer3web version 4.1.0. For amplification of the *virB1* gene, the primers VirB1_for 5′ – GTTACTACGCCGGCAACTTT – 3′ and VirB1_rev 5′ – GCCGCTATAACCTTCCCAGT – 3′ were used. For the *virB2* gene the primers VirB2_for 5′ – CTCATTGTCTCCATCGCTGC – 3′ and VirB2_rev 5′ – GATGACAAGCGGGATCACAG – 3′ were used. For the *virB10* gene the primers VirB10_for 5′ – AGACAAGACGATGGTGCAGA – 3′ and VirB10_rev 5′ – GAGAGTACCCAGCGATCCAG – 3′ were used. For all reactions, the thermal profile consisted of initial denaturation and enzyme activation for 2 min at 95 °C, followed by 35 cycles at 94 °C for 30 s, 55 °C for 30 s, and 72 °C for 30 s, with a final extension at 72 °C for 7 min. PCR products were verified by automated electrophoresis performed with the instrument QIAXcel (QIAGEN) and purified by the QIAquick PCR purification kit (QIAGEN) for DNA sequencing with the BigDye Terminator V1.1 Cycle Sequencing kit. Sequencing products were purified by the DyeEX 2.0 spin kit (QIAGEN) and analyzed by capillary electrophoresis with a 3500 Genetic Analyzer (Life Technologies) equipped with a 50 cm long capillary filled with the separation medium POP-7 polymer. Sequences were analyzed by the SeqStudio Genetic Analyzer software and the Sequence Scanner Software v2.0 (ThermoFisher).

## 3. Results

### 3.1. MucR Binds the Promoters of babR and virB Genes

**Figure 1:**
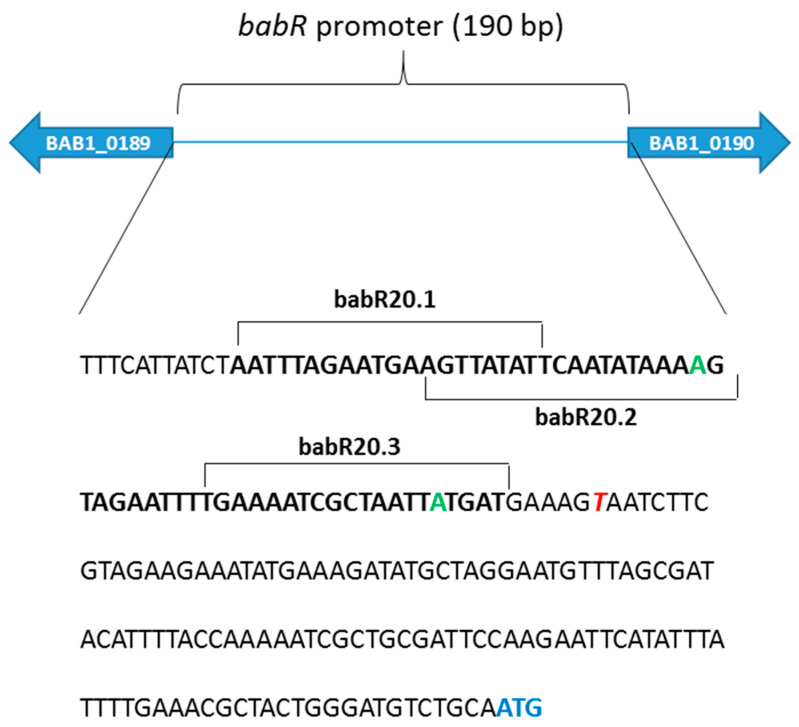
Schematic representation of *babR* promoter characterized by Caswell C.C. et al. [22]. Green indicates the position at ‒35 and ‒10 from the transcription starting site which is indicated in italic red. The ATG of the *babR* gene is reported in blue. The sequence of babR60 used in the electrophoretic mobility shift assays (EMSAs) of MucR is in bold. The sequences of the three 20 bp oligonucleotides tested as MucR target sites are also indicated (babR20.1, babR20.2 and babR20.3).

The gene encoding BabR has been shown to be strongly repressed by MucR in both *B. abortus* 2308 [4,14] and *B. melitensis* 16M [33]. The promoter of the *B. abortus babR* gene has been characterized in detail by Caswell et al. [22]. Its sequence is AT-rich and presents multiple T–A steps. To investigate whether MucR can bind the *babR* promoter, we designed a 60 bp oligonucleotide, named babR60, whose sequence was derived from the *babR* promoter and spans from the nucleotide at -65 to the nucleotide at -5 from the transcription starting site which is located 119 nucleotides upstream of the *babR* start codon (Figure 1). 

**Figure 2:**
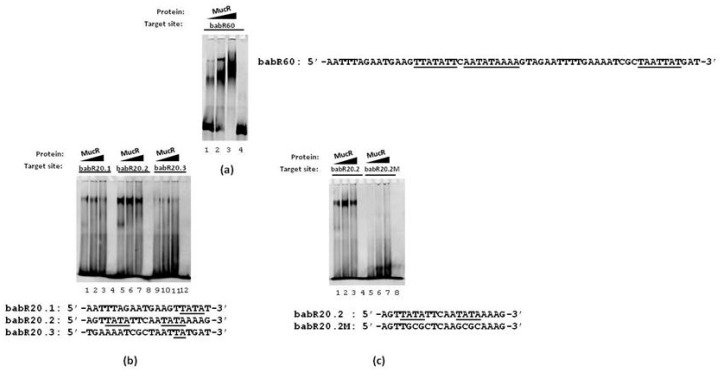
MucR binds the *babR* promoter. EMSAs of MucR with (**a**) babR60 oligonucleotide, (**b**) babR20.1, babR20.2 and babR20.3 oligonucleotides, (**c**) babR20.2 and the mutant version babR20.2M. In (**a**–**c**) the growing amount of protein used is indicated on the top of the lanes and the single-stranded sequences of the double-stranded oligonucleotides tested as target sites of MucR are reported. The AT-rich regions comprising T–A steps are underlined. In panel (**a**), lane 4 shows the sample containing only babR60. In panel (**b**), lanes 4, 8 and 12 show samples containing only babR20.1, babR20.2 and babR20.3, respectively.

The double-stranded oligonucleotide babR60 was used as a target site of MucR in EMSA. The results show that MucR is able to bind babR60 (Figure 2a) demonstrating that the *babR* promoter is a target of MucR in *B. abortus*. 

We have recently reported that MucR could be a histone-like (H-NS) protein able to form higher-order oligomers and recognizing AT-rich sequences containing T–A steps like the H-NS proteins present in β- and γ-proteobacteria [13,14,34]. To gain a clearer picture of the individual contributions of specific AT-rich regions within the *babR* promoter to MucR binding, we designed three 20 bp oligonucleotides, named babR20.1, babR20.2 and babR20.3, whose sequences represent overlapping segments of babR60 (Figure 1 and Figure 2b). The three 20 bp oligonucleotides sequences are 85% AT-rich and each of them contains T–A steps (Figure 2b). We tested babR20.1, babR20.2 and babR20.3 as target sites of MucR in EMSAs and we found that MucR binds all the three 20 bp oligonucleotides tested (Figure 2b). Importantly, MucR appears to bind most strongly to babR20.2 which contains T–A steps spread along its sequence. We designed a mutant version of babR20.2, named babR20.2M, in which thymine and adenine bases constituting T–A steps were substituted by guanine and cytosine bases and we tested this oligonucleotide as a target site of MucR in EMSAs. The babR20.2M failed to bind MucR (Figure 2c) demonstrating that T–A steps mutated in this oligonucleotide play an important role in the MucR interaction with *babR* promoter.

**Figure 3:**
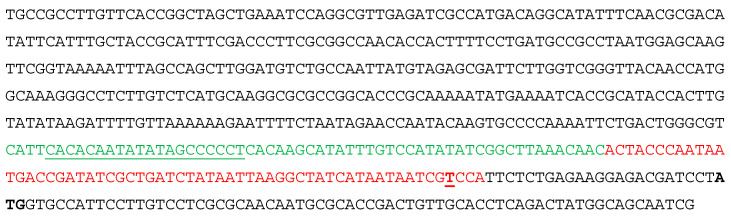
The promoter region of *virB* operon. The sequence reported spans from the thymine occupying the position 67,081 on chromosome II of *B. abortus* 2308 to the guanine in position 70 in the *virB1* gene sequence. The thymine constituting the transcription starting site is in bold red and underlined. The oligonucleotides vir60.1 and vir60.2 tested as DNA target sites of MucR are in green or red, respectively, while the 20 bp oligonucleotides vp1 is underlined.

The protein BabR is a repressor of the *virB* operon. MucR has been shown to have a positive impact on *virB* gene expression in *B. melitensis* 16M [33], but the *virB* genes were not detected in the *B. abortus* 2308 MucR regulon in the studies described by Caswell et al. [4]. The expression patterns of *virB* genes are known to be different in *B. abortus* and *B. melitensis* and exposure to specific environmental stimuli is required to observe expression of these genes in *B. abortus* 2308 [22,35]. Thus, the possibility exists that an impact of MucR on *virB* expression in *B. abortus* 2308 was not detected in the study described by Caswell et al. due to the experimental conditions employed (i.e., routine cultivation in brucella broth). In this scenario, we decided to investigate whether MucR could also interact with the *virB* operon promoter with the aim of disclosing a possible double role played by MucR interacting with both the *babR* promoter and *virB* operon promoter. To determine whether the *virB* genes are direct targets of MucR regulation in *B. abortus* 2308, we analyzed the sequence spanning from nucleotide position 67,081 to 67,583 on chromosome II of this bacterium’s genome (Figure 3). 

**Figure 4:**
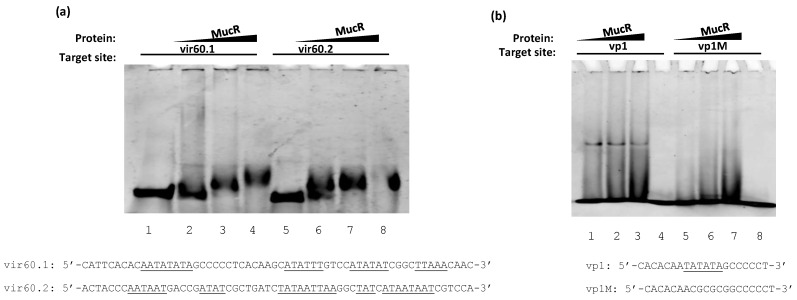
MucR binds the *virB* operon promoter. EMSA of MucR with (**a**) the 60 bp oligonucleotides vir60.1 and vir60.2, and (**b**) with the 20 bp oligonucleotides vp1 and vp1M. In (**a**–**b**) the single-stranded sequence of the double-stranded oligonucleotides tested as target sites of MucR are reported. The AT-rich region containing T–A steps are underlined. The growing amount of protein used is reported on the top of the lanes. In panel (**a**), lane 1 shows the sample containing only vir60.1 and lane 5 the sample containing only virB60.2. In panel (**b**), lane 4 shows the sample containing only vp1 and lane 8 the sample containing only vp1M.

This region contains the well-characterized *virB1* promoter [22,36] and the region starting at -143 bp from the ATG start codon of *virB1* comprises AT-rich sequences containing multiple T–A steps. We designed two 60 bp oligonucleotides, the first one named vir60.1 starting at ‒143 bp from the *virB1* ATG start codon and the second one, named vir60.2, starting at ‒83 bp from the *virB1* ATG (Figure 3). We then examined the interactions of these two oligonucleotides with MucR in EMSAs. The results demonstrate that MucR can bind both the two 60 bp oligonucleotides tested (Figure 4a). To confirm that MucR binding sites are AT-rich sequences containing T–A steps also in the *virB* promoter, we carried out EMSAs of MucR using as target sites a 20 bp oligonucleotide named vp1, whose sequence is included in vir60.1 oligonucleotide, and a mutant of vp1 named vp1M, in which thymine and adenine bases constituting T–A steps were substituted by GC bases (Figure 4b). The results demonstrate that MucR can bind vp1 but fails to bind vp1M, indicating that AT-rich sequences containing T–A steps also represent important MucR binding sites in the *virB* promoter. In all the EMSAs carried out with 20 bp oligonucleotides, the highest amount of protein used does not result in more bound DNA. This is in agreement with what we observed with the other proteins of the Ros/MucR family and it is likely due to the aggregation propensity of these proteins being able to form higher-order oligomers [12,37].

**Figure 5:**
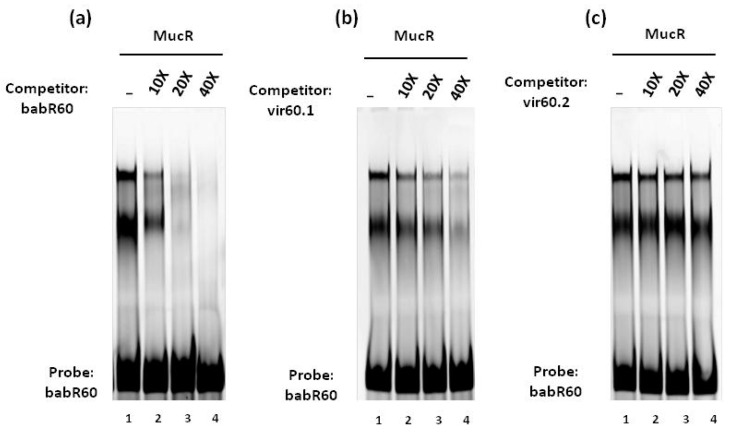
MucR binds with higher affinity to its target site in the babR promoter than it does to its target site in the virB promoter. Competition assays of MucR binding to babR60 using (**a**) babR60, (**b**) vir60.1 and (**c**) vir60.2 as competitors. The amount of excess of each competitor compared to the amount of probe is indicated at the top of the lanes.

We previously proposed that MucR binds to DNA via low specificity interactions with AT-rich regions in a manner reminiscent of H-NS [13,14]. H-NS demonstrates a broad range of affinities for AT-rich regions, and the capacity of this protein to function as a transcriptional regulator is strongly influenced by its affinity for specific DNA-target sites [38]. To investigate whether MucR binds with different affinity to the 60 bp target site babR60, which was identified in the promoter of *babR*, compared to the 60 bp target sites vir60.1 and vir60.2 which are present in the *virB* promoter, we carried out competition experiments with these oligonucleotides in EMSAs. The results show that MucR binds babR60 with higher affinity compared to vir60.1 and vir60.2. The results also show that vir60.1 is a better MucR target site than vir60.2 (Figure 5). 

The oligonucleotide babR60 is more AT-rich (83% of A and T bases) than vir60.1 and vir60.2 which share a similar percentage of adenine and thymine (60.1 is 67% AT-rich and 60.2 is 69%). Furthermore, the arrangement of A and T bases is different in these three oligonucleotides forming stretches of T–A steps only in babR60 and vir60.1. 

Since the interaction of MucR with the promoters of *babR* and *virB* genes was a focus of this study, we decided to analyze the sequences of the *mucR* gene and that of the *babR* and *virB* gene promoter regions in 18 *B. abortus* strains. These strains were isolated from water buffaloes bred in southern Italy, in a region characterized by brucellosis endemicity [39,40], so we were able to evaluate the presence of mutational events. We also extended our genome sequencing analysis to the *babR* and *virB* genes. No mutations were found in any of the promoter regions or in the *mucR* and *babR* genes. Analysis of the *virB* genes revealed that genomic sequences of the field strains harbored point mutations in *virB1, virB2, virB5* and *virB10* compared to the reference strain 2308. 

The point mutation G593A found in the *virb5* gene was present in all the analyzed strains, while the mutations found in *virB1, virB2* and *virB10* turned out to be associated with the biovar of the *Brucella* strain. Indeed, the C299T nucleotide substitution in the *virB10* gene was associated to *B. abortus* biovar 1, while the nucleotide substitutions A622G in *virB1* and G298T in *virb2* appeared associated with the biovar 3. The presence of these biovar-associated mutations was verified by DNA sequencing of *virB1*, *virB2* and *virB10* genes in 68 *B. abortus* field strains belonging to the Experimental Zooprophylactic Institute of Southern Italy bacterial collection. Sequence analysis confirmed the presence of the *virB10* C299T mutation in all biovar 1 strains tested, and the *virB1* A266G mutation in all biovar 3 strains analyzed (Fisher’s exact test two-tailed *p* value < 0.0001; Supplementary Table S1). The *virB2* G298T mutation instead appeared variably distributed among analyzed strains indicating that this mutation is not associated with either *B. abortus* biovar 1 or 3. The mutation C299T that we detected in the *virB10* gene was also found in the *B. abortus* strain 104M, a spontaneously attenuated strain, used as a vaccine strain in humans for decades in China [41]. 

### 3.2. MucR Has Minimal Impact on the Expression of the virB Genes in *B. abortus* 2308

**Figure 6:**
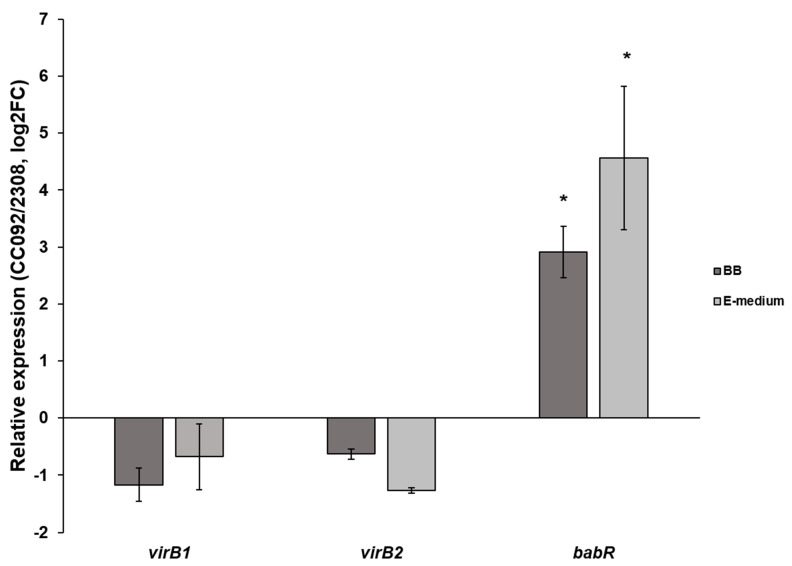
Relative expression of *virB1*, *virB2* and *babR* in *B. abortus* 2308 (solid bars) and CC092 (hashed bars) after growth in Brucella broth (BB) or upon exposure to a virulence-inducing condition (E-medium). Asterisks indicate significant difference in relative expression (*p* value < 0.05) as determined by one sample t-test. The *p* value in the case of bars without asterisk is >0.05.

As noted previously, MucR repression of *babR* expression has been demonstrated in both *B. abortus* 2308 and *B. melitensis* 16M and the results presented here indicate that this repression is a direct effect of MucR binding to the *babR* promoter. To determine the effect of MucR binding on the expression of the *virB* genes, *B. abortus* 2308 and the isogenic *mucR* mutant CC092—which was obtained by introducing an in-frame *mucR* deletion into *B. abortus* 2308 [4]—were grown in Brucella broth and E medium buffered at pH 4.5, and the relative expression patterns of the *virB1 and virB2* genes were evaluated. The pH 4.5 cultivation condition in E medium effectively induces *virB* expression in *B. abortus* 2308 [22]. As shown in Figure 6, no significant difference in expression of the *virB1* and *virB2* genes were detected between *B. abortus* 2308 and the *mucR* mutant strain CC092 following cultivation either in Brucella broth or E medium at pH 4.5. In contrast, the expression of *babR* was significantly higher in the *B. abortus mucR* mutant strain than it was in the parental 2308 strain following growth in both media. These experimental findings suggest that MucR has a limited impact on the expression of the *virB* genes in *B. abortus* 2308. 

## 4. Discussion

The protein MucR belongs to the Ros/MucR family whose studied members are all involved in the regulation of the expression of genes necessary to successfully infect the eukaryotic host or to establish a symbiosis with it [4,19]. The ability of the protein Ros from *Agrobacterium tumefaciens* to regulate the expression of *vir* genes was deeply investigated [10] and the role of MucR from *B. melitensis*, *B. abortus* or *S. meliloti* in regulating the expression of genes which are crucial for infectious or symbiotic processes is well documented [4,5,19]. In *C. crescentus*, the protein MucR is also involved in the cell cycle progression [42] and notably the *Brucella* cell cycle has been linked to this bacterium’s pathogenic potential [43]. Recently, we have demonstrated that MucR can form higher-order oligomers and binds AT-rich sequences containing T–A steps [12,13,14]. These findings led us to compare the role of MucR in the α-proteobacteria to that of H-NS proteins that regulate the expression of virulence genes in β- and γ-proteobacteria. The way adopted by H-NS to regulate gene expression is to bind AT-rich sequences and to use these targets as nucleation sites for extending their presence on the nucleoid by oligomerization [15,16]. The number of genes regulated by H-NS is pretty wide and it is hypothesized that they provide an advantage for bacteria in need to repress pathogenicity islands acquired by horizontal gene transfer when infection cannot occur [44,45,46]. Furthermore, H-NS proteins repress genes whose products can have unwelcome effects on the physiology of the cell when they are not necessary [47]. H-NS proteins have never been found in many species of α-proteobacteria [27] and we proposed that MucR could be the H-NS-like protein that has never been identified in *Brucella* [13,14]. We showed the ability of MucR to bind scrambled AT-rich sequences containing T–A steps and to share this ability with Ml proteins from *M. loti* [12]. We also showed that MucR from *B. abortus* is a heat resistant protein and can bind its own promoter, recognizing multiple binding sites just like the H-NS proteins [13]. The expression of many genes involved in the infection of the eukaryotic host are regulated by MucR [4], but the ability of this protein to bind the promoters of most of the regulated genes has not been investigated so far. One of the gene whose expression is regulated by MucR is *babR* which encodes for a LuxR-like regulator playing a major role in repressing *virB* genes and working in concert with the VjbR protein to coordinate proper *virB* gene expression [18,21,22]. The first 11 *virB* genes play a crucial role in the infection process [48] because they encode for proteins assembling the T4SS complex which is used by many bacteria to translocate molecules which are essential to infect the host [24,25]. In this study, we demonstrate for the first time the ability of MucR to bind the promoter of the *babR* gene. This finding clarifies the direct role of MucR in *babR* gene repression. The modality adopted by MucR to bind the *babR* promoter is the same as for binding its own promoter recognizing AT-rich multiple binding sites containing T–A steps [13]. This is in line with our hypothesis that this protein is the H-NS-like protein that has never been found before in *Brucella* or in other α-proteobacteria. In fact, we have clearly demonstrated in this study that MucR recognizes multiple AT-rich DNA-target sites containing T–A steps in the *babR* promoter. Together with MucR ability to oligomerize [14], the modality of MucR to bind the promoters studied suggests that the AT-rich DNA-targets are nucleation sites from which this protein can stretch its presence and keep repressed target genes. 

In *B. melitensis*, a strain lacking the *mucR* gene shows an upregulation of *virB* genes indicating a positive impact of MucR protein on *virB* gene expression [33]. *virB* genes are not among targets of MucR identified by Caswell et al. [4]. Since the expression of *virB* genes is induced by different environmental stimuli, it was still possible that MucR has an impact on *virB* gene expression regulation and that the role of this protein in regulating *virB* gene expression was not disclosed under the condition used by Caswell et al. [4]. In this study, we have investigated the ability of MucR to bind the promoter of *virB* genes and we have analyzed the expression level of *virB1* and *virB2* in *B. abortus* CC092 lacking the *mucR* gene. The results show that MucR can recognize some AT-rich DNA-targets in the *virB* promoter, but the expression level of *virB1* and *virB2* does not change significantly in the *B. abortus* CC092 strain compared to the wild-type strain 2308 under all conditions tested. When analyzing the expression level of the *babR* gene in *B. abortus* CC092, a strong upregulation is detected in comparison to the *babR* expression in the *B. abortus* wild-type strain 2308. In EMSAs, the affinity of MucR binding to DNA-targets in the *virB* promoter is low when compared to that of the binding to the *babR* promoter target sites. Altogether, our findings suggest that MucR plays a major role in repressing the *babR* gene whose product is crucial for *virB* gene expression regulation. Our results further support our previous hypothesis that MucR is an H-NS-like protein. In fact, H-NS proteins exert a direct role in repressing genes by binding directly to promoters presenting high affinity target sites and structuring DNA to prevent the promoter access to transcription machinery [38]. However, the H-NS can modulate their effect on gene expression based on the presence of the number of target sites and the affinity for these sites [38]. MucR tightly binds the *babR* promoter, recognizing multiple binding sites whereas it loosely binds the target sites present in the *virB* promoter so that its effect on the *virB* promoter does not significantly change *virB* expression in *B. abortus* CC092 compared to the 2308 strain. The action of H-NS proteins on the expression of target genes can also be exerted together with other factors [47]. We cannot exclude the possibility that MucR, like H-NS, plays a role in regulating *virB* gene expression together with other protein partners and that the sole absence of this protein in *B. abortus* CC092 is not sufficient to deregulate the *virB* genes. Intriguingly, despite the fact that the BabR repressor of *virB* genes is expressed at a higher level in the CC092 strain, *virB* expression level does not change in the CC092 strain compared to the 2308 strain even when both strains are cultured in E-medium which activates *virB* genes. We can hypothesize that the expression of *virB* genes is under the control of many factors and is stimulated by environmental stimuli. Once activated, more than one factor will be involved in turning off *virB* expression. However, no study to identify MucR protein partners has been carried out so far in *B. abortus* and the results in this paper encourage MucR interactome studies for shedding light on the partners of MucR in gene expression regulation.

Finally, since the interaction of MucR with *virB* and *babR* promoters was a focus of this study, we analyzed the genomic sequences of both promoters and of the *mucR* gene from 18 field isolated *B. abortus* strains and found that they are conserved. We extended our analysis to *babR* and *virB* genes, revealing that mutations were present only in *virB* genes. Particularly, the mutation found in *virB1* and in *virB10* turned out to be associated with *B. abortus* biovar 3 and biovar 1, respectively. The biovar-specific mutations found might contribute to differences in host–pathogen interactions between *B. abortus* biovar 1 and 3. Although the spillover of *Brucella* species and biovars into a wide range of animal species is well known, a host preference can still be reported. Indeed, in a previous study on the occurrence of *B. abortus* in domestic ruminants in southern Italy, biovar 1 was significantly associated with water buffalo, while biovar 3 was significantly associated with cow [49]. 

## 5. Conclusions

Our findings in this paper demonstrate for the first time that MucR from *B. abortus* plays a direct role in regulating the expression of the Lux-like type regulator BabR by binding multiple target sites in the *babR* promoter. Our in vitro experiments also demonstrate that MucR can bind the *virB* promoter, but with a lower affinity compared to *babR* promoter binding. RT-qPCR analysis of *babR* and *virB* gene expression in *B. abortus* strain CC092 defective of *mucR* compared to the wild-type strain 2308 showed that MucR exerts a strong effect on *babR* expression and seems to have no effect on that of *virB* genes. The findings in this paper support the hypothesis that MucR is an H-NS-like protein and suggests future studies should consider this new data about MucR and Ros/MucR family members in light of the new findings reported in our study.

## Supplementary Materials

The following are available online at https://www.mdpi.com/2218-273X/10/5/788/s1, Table S1: Mutations associated to *B. abortus* biovar found in virB1 and virB10 from field isolated strains are reported.
