# Supplementary material for: Different Impacts of MucR Binding to the babR and virB Promoters on Gene Expression in Brucella abortus 2308
Source: Biomolecules. 2020 May 19;10(5):788. doi: 10.3390/biom10050788 (PMC7277663; doi:10.3390/biom10050788)
Supplement: Supplementary file 1 [file biomolecules-10-00788-s001.pdf]

Supplementary Materials

Different Impacts of MucR Binding to the *babR* and *virB* Promoters on Gene Expression in *Brucella abortus* 2308

Giorgia Borriello[1#], Veronica Russo[2#], Rubina Paradiso[1], Marita Georgia Riccardi[1], Daniela Criscuolo[1], Gaetano Verde[3,4], Rosangela Marasco[2], Paolo Vincenzo Pedone[2], Giorgio Galiero[1], Ilaria Baglivo[2*].

[1] Experimental Zooprophylactic Institute of southern Italy, via Salute, 2, 80055, Portici, Italy

[2] Department of Environmental, Biological and Pharmaceutical Sciences and Technologies , University of Campania "Luigi Vanvitelli", via Vivaldi - 43, Caserta, 81100, Italy

[3] Institute of Genetics and Biophysics (IGB) "Adriano Buzzati-Traverso", Consiglio Nazionale delle Ricerche (CNR), Naples, 80134, Italy.

[4] Flomics Biotech, Carrer Dr. Aiguader 88, 08003 Barcelona, Spain

* Correspondence: ilaria.baglivo@unicampania.it; Tel.: 0039 0823 274598 (I.B.); giorgio.galiero@cert.izsmportici.it; Tel. 0039 081 7865201 (G.G.)

# These two authors contributed equally as first authors of this study.

Table S1. Mutations associated to *B. abortus* biovar found in virB1 and virB10 from field isolated strains are reported.

Mutation in *virB* genes (position is given on the base of the coding sequence from *B. abortus* 2308)	*B. abortus* strain and accession number of genomic sequence	Biovar associated to mutation
virb1: A622 ➔G	**15500** (CP023231; CP023232)<br>**21614** (CP023233; CP023234)<br>**67761** (CP023223; CP023224)<br>**72871** (CP023239; CP023240)<br>**21630** (NZ_CP023235; NZ_CP023236)<br>**38127** (NZ_CP023237; NZ_CP023238)<br>**149279** (NZ_CP023227; NZ_CP023228)<br>**69103** (NZ_CP023225; NZ_CP023226)<br>**57750** (NZ_CP023221; NZ_CP023222)<br>**49188** (NZ_CP023217; NZ_CP023218)	3
virb10: C299➔ T	**84573** (NZ_CP023241; NZ_CP023242)<br>**9510 (**NZ_CP023308; NZ_CP023309);<br>**7863** (NZ_CP023229; NZ_CP023230)<br>**14330** (CP023243; CP023244)<br>**28375** (NZ_CP023211; NZ_CP023212)<br>**33295** (CP023213; CP023214)<br>**49839** (NZ_CP023219; NZ_CP023220)<br>**40046** (NZ_CP023215; NZ_CP023216)	1
